# An *en masse *phenotype and function prediction system for *Mus musculus*

**DOI:** 10.1186/gb-2008-9-s1-s8

**Published:** 2008-06-27

**Authors:** Murat Taşan, Weidong Tian, David P Hill, Francis D Gibbons, Judith A Blake, Frederick P Roth

**Affiliations:** 1 Department of Biological Chemistry and Molecular Pharmacology, Harvard Medical School, Longwood Avenue, Boston, Massachusetts 02115, USA; 2 Computational Biology and Bioinformatics, The Jackson Laboratory, Main Street, Bar Harbor, Maine 04609, USA; 3 Merrimack Pharmaceuticals, Kendall Square, Cambridge, Massachusetts 02139, USA

## Abstract

**Background:**

Individual researchers are struggling to keep up with the accelerating emergence of high-throughput biological data, and to extract information that relates to their specific questions. Integration of accumulated evidence should permit researchers to form fewer - and more accurate - hypotheses for further study through experimentation.

**Results:**

Here a method previously used to predict Gene Ontology (GO) terms for *Saccharomyces cerevisiae *(Tian *et al*.: Combining guilt-by-association and guilt-by-profiling to predict *Saccharomyces cerevisiae *gene function. *Genome Biol *2008, 9(Suppl 1):S7) is applied to predict GO terms and phenotypes for 21,603 *Mus musculus *genes, using a diverse collection of integrated data sources (including expression, interaction, and sequence-based data). This combined 'guilt-by-profiling' and 'guilt-by-association' approach optimizes the combination of two inference methodologies. Predictions at all levels of confidence are evaluated by examining genes not used in training, and top predictions are examined manually using available literature and knowledge base resources.

**Conclusion:**

We assigned a confidence score to each gene/term combination. The results provided high prediction performance, with nearly every GO term achieving greater than 40% precision at 1% recall. Among the 36 novel predictions for GO terms and 40 for phenotypes that were studied manually, >80% and >40%, respectively, were identified as accurate. We also illustrate that a combination of 'guilt-by-profiling' and 'guilt-by-association' outperforms either approach alone in their application to *M. musculus*.

## Introduction

With the ever-increasing collection of high-throughput experimental techniques, data acquisition at the genomic scale has never occurred more rapidly. As the raw data continue to amass, each biologist is faced with the difficult challenge of integrating and interpreting the data that are most relevant to each specific research question. Comprehensive annotation systems are thus of paramount importance, as evidenced by the integration of a large number of data types in many model organism databases [1-4]. Such databases are now the researcher's starting point for informed hypothesis generation, making the daunting task of curating the source data for representation in model organism databases crucial to effective science.

Recognizing this problem, curation systems are becoming increasingly reliant on computational approaches to assist in the annotation process. Sequence similarity (both at the nucleotide and peptide levels) has traditionally been the primary source of automated annotation. Particular motifs found within a sequence can be used to infer a gene product's molecular activity, with increasing work being done to identify the domains that facilitate protein interactions [5]. Similarly, when working with an uncharacterized gene, identifying characterized homologous sequences in other species is often the first step to understanding the gene of interest (which underscores the great benefit of multiple model organism and associated databases). Identifying gene function solely through sequence-based approaches has its limitations, however. Often sequence similarity must be restrictively high for correct inference of functional annotation [6], and in many cases no homolog has yet been characterized.

The integration of other high-throughput data types has been shown to be effective for combating the limitations of annotation systems reliant only on sequence similarity [7-13]. Recently, an attempt has been made to assess the ability of such methods to make Gene Ontology (GO) annotations in *Mus musculus*: the MouseFunc project [14]. A wide range of data was collected for each of 21,603 mouse genes, and 9 models were built by research groups working independently to determine whether the successes achieved with previous data integration methods on single-celled eukaryotes could be replicated in a complex mammalian system with a large fraction of genes being wholly (or incompletely) un-annotated. Here we present results from one of the nine modeling approaches submitted for the MouseFunc project (the methodology as applied to *Saccharomyces cerevisiae *is described in [15]), producing an updated set of genome-wide annotation predictions for approximately 3,000 GO terms [16] for *M. musculus*. In addition, we provide predictions for approximately 4,000 mammalian phenotype terms [17], which can assist in selection of (often resource-intensive) phenotyping assays for knockout mice. We evaluate classifier performance according to genes not used in training the predictive models. A subset of the highest confidence novel predictions are investigated in the published literature and are discussed further.

## Results

### Compilation of data

Data of a large number of distinct types were collected and organized for both phenotype and GO term prediction. Protein domain annotations, protein-protein interactions, expression data, disease data, and phylogenetic profile data for 21,603 *M. musculus *genes were taken from the dataset established for the MouseFunc project [14]. Expanded and more recent (relative to the MouseFunc data [14]) sets of mammalian phenotype and GO term annotations for the same mouse genes were acquired from Mouse Genome Informatics (MGI) [17]. Detailed descriptions of the data organization and pre-processing are given in the Materials and methods (see below).

### A combined learning approach for annotation prediction

A machine-learning approach was used here for function and phenotype prediction. We used a weighted combination of two distinct models, rather than adopting a single family of models for prediction. For clarity, a brief description of the general method is described here, with further details available in this issue [15].

First, we applied a gene-centric 'guilt-by-profiling' approach. Here, properties associated with single genes (for example, matches to specific protein domain patterns) were used to infer various annotations for those genes. In this case, a distinct random forest classifier [18] was constructed for each GO term or phenotype. Second, a paired-gene approach was applied ('guilt-by-association'). Pairs of genes are connected by weighted edges, with weights representing belief that the two genes are functionally linked (that is, a functional-linkage graph [19]). Genes with strong functional links tend to share annotations, and in cases where one member of a functionally linked pair has an annotation and the other does not, a new annotation can be inferred.

Annotation predictions made by each of these two component classifiers were then combined using a regression model that maximizes a chosen performance measure, exploiting the best features of both predictive classifiers. A brief description is given in the Materials and methods (see below) and in more detail in an accompanying paper describing the methodology and its application to *S. cerevisiae *gene function [15].

### Genome-wide application of a combined learner to *M. musculus*

Predictive models for 2,938 GO and 3,914 mammalian phenotype terms were made; 21,603 *M. musculus *genes were given quantitative scores for each possible annotation of each term (for a total of 21,603 × [2,938 + 3,914] ≈ 148 × 10^6 ^prediction scores computed).

#### Subdivision of terms based on specificity

The 2,938 GO terms currently (as of November 2006) annotated with a number of genes in the range [3,300] were selected for training and prediction (here the notation '[*a*, *b*]' indicates the range from *a *to *b*, inclusive of *a *and *b*). We wished to evaluate separately the performance for terms of different types and levels of generality. To this end, terms were divided into 12 disjoint sets, each representing both a single branch in the GO (that is, biological process [BP], molecular function [MF], and cellular component [CC]) and a range in current annotation count, that is, the number of genes annotated with the term (we considered the ranges [3,10], [11,30], [31,100], and [101,300]). The number of terms in each set is given in Table 1. A similar division was created for mammalian phenotype terms (from the Mammalian Phenotype Ontology [17]), and terms having current annotation count in the range [3,300] were selected for prediction. This collection was similarly divided into four sets based on annotation count (as of October 2007) - the number of terms in each set is shown in Table 1.

**Table 1:** Division of 2,938 GO terms and 3,914 phenotype terms into categories

	BP	CC	MF	Phenotype
[3,10]	764	140	448	1,738
[11,30]	565	98	185	1,153
[31,100]	321	77	128	729
[101,300]	142	30	40	294

BP, biological process; CC, cellular component; GO, Gene Ontology; MF, molecular function.

#### Quantitative predictive performance estimates

To evaluate the participating classifiers, the MouseFunc project employed the area under the receiver operator characteristic (ROC) curve (AUC-ROC) and precision at specified levels of recall (precision is the fraction of predictions [scores above a given threshold] that are true, while recall is the fraction of true annotations recovered above some score threshold). We use the same general approach here, but include an additional measure: 'mean average precision' (MAP). An ROC curve indicates the relationship between true positives and false positives as the score threshold for calling a prediction is varied. Also used are points along the precision-recall curve (as the threshold varies), with MAP being the mean precision obtained at all distinct recall levels. In the case of a tie among scores, the average precision over all permutations of the response variable (the GO or phenotype term annotations) is taken as the precision for that level of recall. The MAP has been identified as a good alternative to other precision-recall based statistics. Most notably, it has been deemed more useful than area under the precision-recall curve (AUC-PR) as a measure of comparison between classifiers [20]. We also include aggregate performance measures for categories such that scores for all terms within a single category are pooled.

Table 2 gives - for each category - the aggregate precision for the combined classifier across varying levels of recall, as well as the AUC-ROC. Precision at low recall is usually the driving criterion for biologists, who generally want to have high precision (accuracy) when examining a 'short list' of candidate genes for association with a particular function or phenotype.

**Table 2:** Pooled performance measures for each category

	P01R*	P05R	P10R	P20R	P50R	AUC-ROC
BP [3,10]	0.41	0.23	0.17	0.08	0.002	0.75
BP [11,30]	0.41	0.28	0.22	0.14	0.01	0.79
BP [31,100]	0.57	0.51	0.42	0.30	0.06	0.75
BP [101,300]	0.60	0.49	0.43	0.32	0.09	0.85
CC [3,10]	0.78	0.43	0.45	0.24	0.02	0.85
CC [11,30]	0.72	0.54	0.49	0.38	0.04	0.86
CC [31,100]	0.71	0.65	0.51	0.36	0.04	0.86
CC [101,300]	0.92	0.72	0.59	0.46	0.12	0.87
MF [3,10]	0.65	0.52	0.52	0.48	0.25	0.88
MF [11,30]	0.70	0.68	0.62	0.56	0.34	0.90
MF [31,100]	0.84	0.74	0.66	0.60	0.43	0.93
MF [101,300]	0.86	0.70	0.69	0.61	0.47	0.92
						
Pheno [3,10]	0.10	0.07	0.03	0.003	0.0006	0.70
Pheno [11,30]	0.20	0.13	0.07	0.02	0.003	0.75
Pheno [31,100]	0.31	0.20	0.14	0.07	0.009	0.78
Pheno [101,300]	0.44	0.33	0.25	0.17	0.04	0.78

*P01R is the precision at 1% recall, and the other headers follow this pattern. AUC, area under the curve; BP, biological process; CC, cellular component; MF, molecular function; Pheno, phenotype; ROC, receiver operating characteristic.

**Figure 1:**
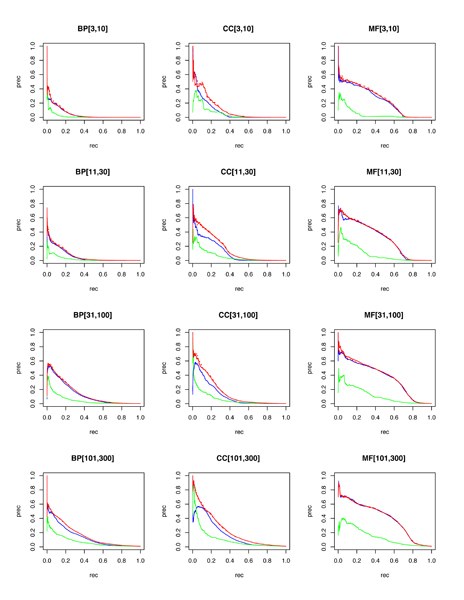
Precision (prec)-recall (rec) plots by pooled GO categories. Random forests are blue, functional linkage trees are green, combined classifier is red (dashed), and the combined/scaled classifier is red (solid). GO, Gene Ontology.

**Figure 2:**
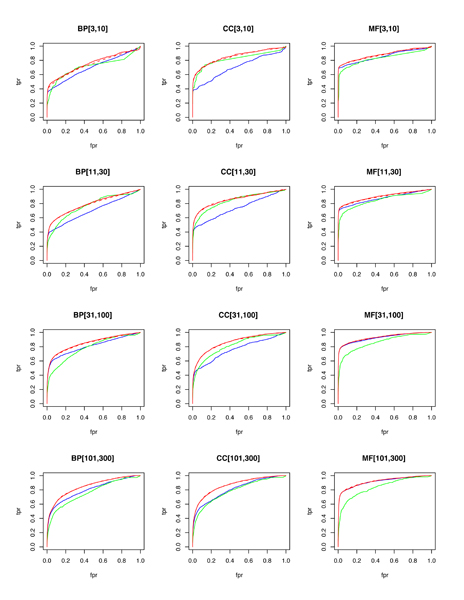
ROC plots by pooled GO categories. Random forests are blue, functional linkage trees are green, combined classifier is red (dashed), and the combined/scaled classifier is red (solid). Fpr, false positive rate; GO, Gene Ontology; ROC, receiver operating characteristic; tpr, true positive rate.

For GO term prediction, mean AUC-ROC for the combined classifier exceeds 0.8 for 9 out of 12 categories (with 0.5 AUC-ROC being expected for random predictions). At 1% recall, the precision ranges from 41% to 92% across the 12 categories, with MF and CC terms being easier to predict for than BP terms, and with difficulty in prediction increasing as the existing annotation count for these terms decreases (due to limited training data availability and a lower prior probability of success due to low prevalence of existing annotations). The performance levels for each category as the threshold varies can be seen in Figures 1 and 2.

**Figure 3:**
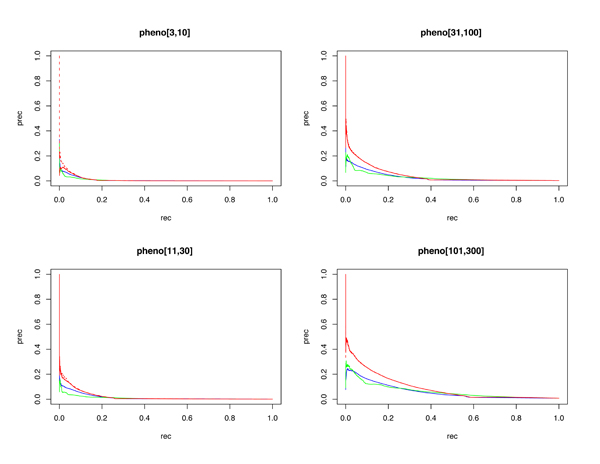
Precision (prec)-recall (rec) plots by pooled phenotype (pheno) categories. Random forests are blue, functional linkage trees are green, combined classifier is red (dashed), and the combined/scaled classifier is red (solid).

**Figure 4:**
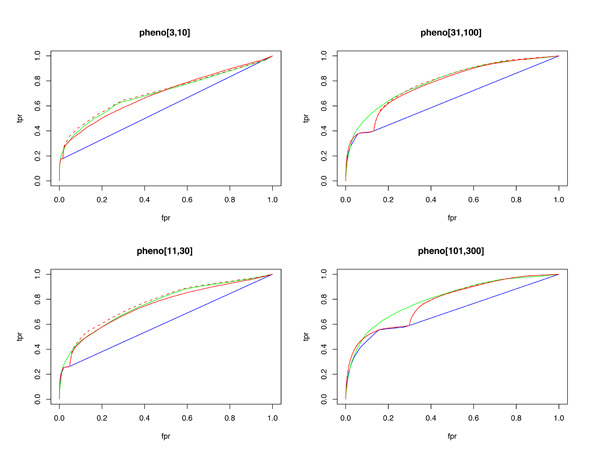
ROC plots by pooled phenotype (pheno) categories. Random forests are blue, functional linkage trees are green, combined classifier is red (dashed), and the combined/scaled classifier is red (solid). Fpr, false positive rate; ROC, receiver operating characteristic; tpr, true positive rate.

Phenotype annotations prove systematically more difficult to classify than GO term annotations (see Discussion, below). The AUC-ROC for each pooled phenotype category exceeds 0.7, with precision at 1% recall ranging between 10% and 44% (again with the observation that terms with lower annotation count have lower predictive accuracy). Performance characteristics along the threshold range are depicted in Figures 3 and 4.

### Examination of top novel predictions

Cross-validation and out-of-bag [18] performance estimates are useful to assess the ability of the classifier to recover the 'gold positives' not used in the training process and, thus, the generalizable performance of the classifier. However, the true potential of such classifiers to impact biology is in their ability to make novel annotations, even for genes used in the training process. Thus, high-scoring 'incorrect' predictions are of extreme interest because they provide novel hypotheses about gene function and may serve to guide choice of experiments. (We note that some predictions may only be 'incorrect' in the sense that cross-validation or held-out test set performance measures consider such cases as 'false positives' because the gene has not yet been annotated with the property, even though such a prediction may in fact be correct and thus 'novel'.) The MouseFunc project introduced a method for prospectively assessing the ability of classifiers to make novel predictions [14]. Training data for MouseFunc reflected the state of annotation knowledge in February 2006. When scores were submitted in October 2006, the classifiers' ability to recover held-out test data was assessed, but in addition the classifiers' ability to predict for annotations that were newly assigned between February and October was measured. The methods in the MouseFunc project (group 'G') are fundamentally the same as those applied here; thus, we expect approximately the same level of novel term prediction performance while using a newer set of GO data for training. It should be noted that the performance reported in that work can be viewed as a pessimistic measure of the actual performance, as only a matter of months elapsed before assessing novel predictions, and many of the apparently false-positive predictions at the end of that period may ultimately be proven correct by future experiments.

To gain intuition from specific examples, we examined some of the most interesting novel predictions within the literature. A gene/term prediction is novel and was considered interesting if it was not currently annotated in the reference database and if there did not exist any current annotation involving the gene and any non-root ancestor of the term. Otherwise, we consider such predictions to be 'refinements' of existing annotations. Within each category, the top interesting gene/term combinations are scanned in decreasing order of confidence (that is, prediction score) and - to avoid over-weighting particular genes or terms - a further filtering step is employed to limit each gene and term to a single appearance in the list to be followed up in depth. This filter is described in detail in the Materials and methods (see below).

#### Literature evaluation of novel GO term predictions

Predicted GO annotations were reviewed by biologists within the MGI group who are experienced with literature curation (DPH and JAB). Three predictions were reviewed for each of the 12 GO categories based on novelty with respect to existing annotations. Each of these 36 predictions were placed in one of the following four classes: class (i), available experimental literature supports the predicted annotation (21 predictions); class (ii), prediction likely to be correct but supported only by indirect evidence in the literature (5 predictions); class (iii), veracity of prediction is unclear (4 predictions); and class (iv), the prediction is incorrect or unlikely to be correct based on current knowledge (6 predictions). We determined that 19 predicted annotations were class (i), that is, they would qualify for annotation by current curation standards but have not yet been annotated. A remaining two had been annotated since our training data were collected, and were, therefore, correct by definition. Excluding 4 'unclear' evaluations, this leads to 26/32 ≈ 81% accuracy. Additional data file 1 lists these predictions, ratings, and evidence supporting the ratings.

Some of the annotations in the first class could be assigned to even more specific terms than the predicted annotation based on current literature. For example, *Adra2*, predicted for annotation with 'blood pressure regulation' (GO:0008217), could be annotated to 'baroreceptor feedback regulation of blood pressure' (GO:0001978), which is a child of 'blood pressure regulation' [21].

In some cases a biological explanation for the evidence that proved useful in making the prediction was not immediately obvious. In such cases, an examination of the reasons for the prediction may shed light on underlying mechanisms of action of the genes involved. An example of this is the class (ii) prediction of subcellular localization of MYBPC1 (myosin binding protein C, slow type) to the A-band. The primary annotations of *Mybpc1* leading to this prediction are protein sequence patterns found by the classifier to be associated with A-band localization (fibronectin type III domains and immunoglobulin I-set domains). Annotations of these domains do not specify any particular localization signal, but the combination of these domain families nevertheless gives rise to A-band annotation predictions. This particular prediction was found likely to be true in our follow-up due to literature-based support [22]. Thus, it may be useful to annotate this combination of Interpro and Pfam domains as being associated with A-band localization.

As one illustrative example of a class (iv) prediction, the gene product of the *Srd5a1 *gene was predicted to be involved in the process of 'genitalia development'. The literature reports that a mutation in this gene product causes defects in parturition, in particular, the cervix of the pregnant female fails to ripen, resulting in the defect [23]. Although cervical ripening is a process that involves the internal genitalia of the female mouse, the cervix is mature before ripening and, therefore, the ripening process itself is not part of the development of the cervix. The process of cervical ripening is instead a process that would be part of 'maternal process involved in parturition' (GO:0060137). In this case, the GO prediction was related, but subtle curator judgment was required to deem the prediction incorrect. There was no other evidence in the MGI literature collection that supported an annotation of *Srd5a1 *to 'genitalia development'.

Another illustrative example from class (iv) was the *Vcl *gene product, which was predicted to be part of the extracellular matrix. The protein has been shown to be part of adhesion contacts that interact with the matrix, but curators could find no evidence that it is part of the matrix itself (for example see work by Ben-Ze'ev and coworkers [24]).

#### Phenotype term predictions

The top 40 phenotype predictions (across all four categories) were also evaluated through an examination of the relevant literature (by DPH and JAB). Of these, 13 were deemed 'would be annotated' or 'likely to be true' (class [i]), 11 were considered 'unclear' (class [iii]), and 16 were found 'unlikely' or 'very unlikely' (class [iv]). Excluding 'unclear' evaluations, the success rate was 13/29 ≈ 45%. Additional data file 2 lists these predictions and evidence supporting the evaluations.

In the case of the enlarged heart phenotype prediction for *Nfatc2*, the absence of an embryonic heart phenotype had been reported [25]. This highlights one of the difficult tasks in predicting and evaluating phenotypes, in that this description does not rule out a heart phenotype if the gene product were perturbed by a different mutation or at another stage of development. Ten phenotype predictions were classified as unclear either because little was known about the gene products, or because the phenotypes that had been reported for knockouts in the genes are severe and might mask the predicted phenotype. Examples of this are *Tbx4 *and *Acvr1b*. Animals with mutations in these genes exhibit severe defects in early embryogenesis that may mask phenotypes such as fused dorsal root ganglia or mandible hypoplasia that would only be revealed by perturbing these genes later in development or perhaps through more subtle mutations [26,27]. In general, phenotype predictions are much more difficult to assess because a phenotype must often be specifically sought or it can be missed.

The value in phenotype prediction lies in its abilities to suggest the most relevant phenotype tests to apply to organisms in which a given gene has been mutated. Many phenotypic assays are resource intensive and are, therefore, unlikely to be performed in the absence of a strong *a priori *hypothesis. An example of a verified class (i) prediction that had not been annotated previously is our association of the *Vav2 *oncogene with abnormal circulating adrenaline levels. *Vav2 *knockout mice have shown defects in heart, arterial walls, and kidneys, as well as tachycardia and hypertension, each associated with adrenaline regulation [28]. Thus, phenotype predictions are often specific and may justify the investment of experimental resources in potentially costly assays (for example, the use of an enzyme-linked immunosorbent assay [ELISA] to measure adrenaline levels in the *Vav2 *knockout mice) that may help pinpoint phenotype and (in some cases) disease etiology.

### Availability of predictions

All predictions have been made available through the worldwide web through a simple database gateway [29].

## Discussion

### Success of a combination-of-learners approach

'Guilt-by-profiling' and 'guilt-by-association' methods have both been previously employed for functional prediction. The combination of these approaches has been applied to the unicellular eukaryote *S. cerevisiae *[13,15]. Here we show that an approach that combines classifiers of both types by logistic regression is superior to that of either base classifier alone in the mammal *M. musculus *for both function and phenotype. A detailed discussion of this combination approach is provided in [15]. Briefly, a single free parameter *α *weighting the contribution of each base classifier was chosen to maximize AUC-PR. To avoid over-fitting, a single *α *was found for each pooled category. Performance evaluation of the algorithm as applied to *M. musculus *function and phenotype is described below.

**Figure 5:**
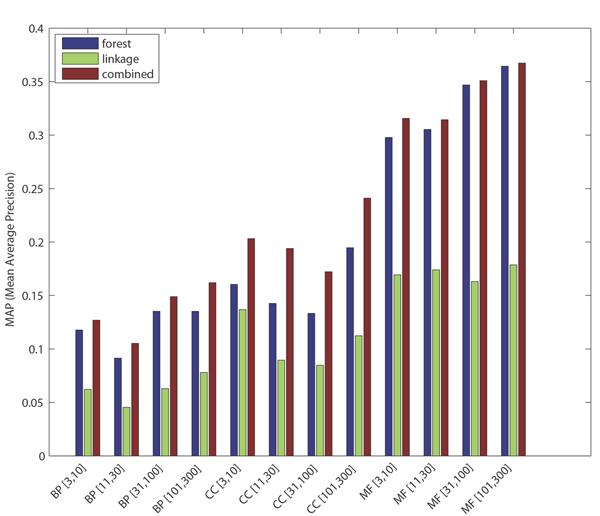
Mean AUC-ROC score across terms within each GO category. AUC, area under the curve; GO, Gene Ontology; ROC, receiver operating characteristic.

**Figure 6:**
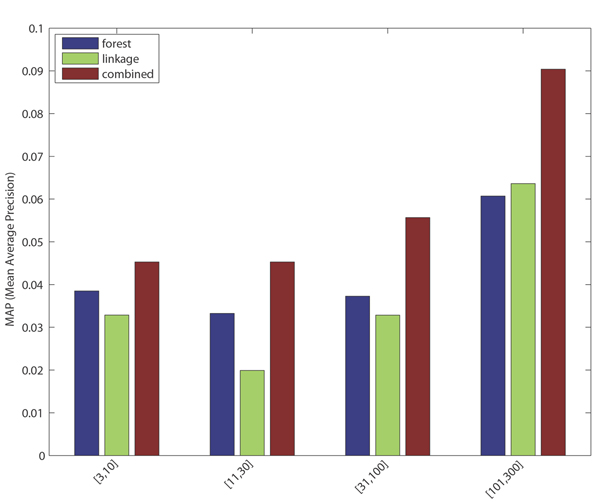
Mean AUC-ROC score across terms within each phenotype (pheno) category. AUC, area under the curve; ROC, receiver operating characteristic.

**Figure 7:**
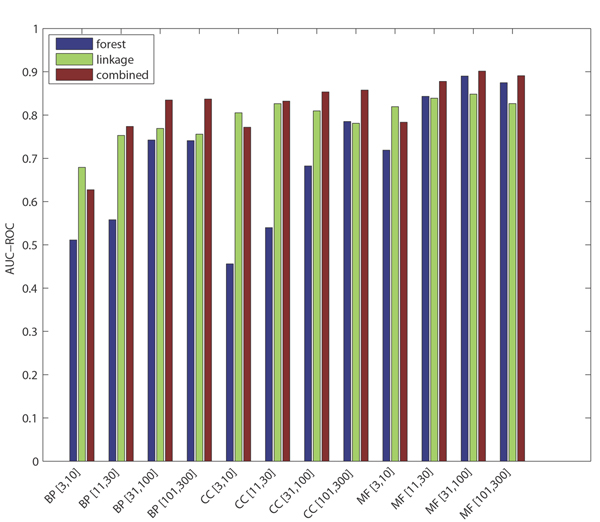
Mean MAP score across terms within each GO category. Note that in all categories the combined score provides a higher mean MAP. GO, Gene Ontology; MAP, mean average precision.

**Figure 8:**
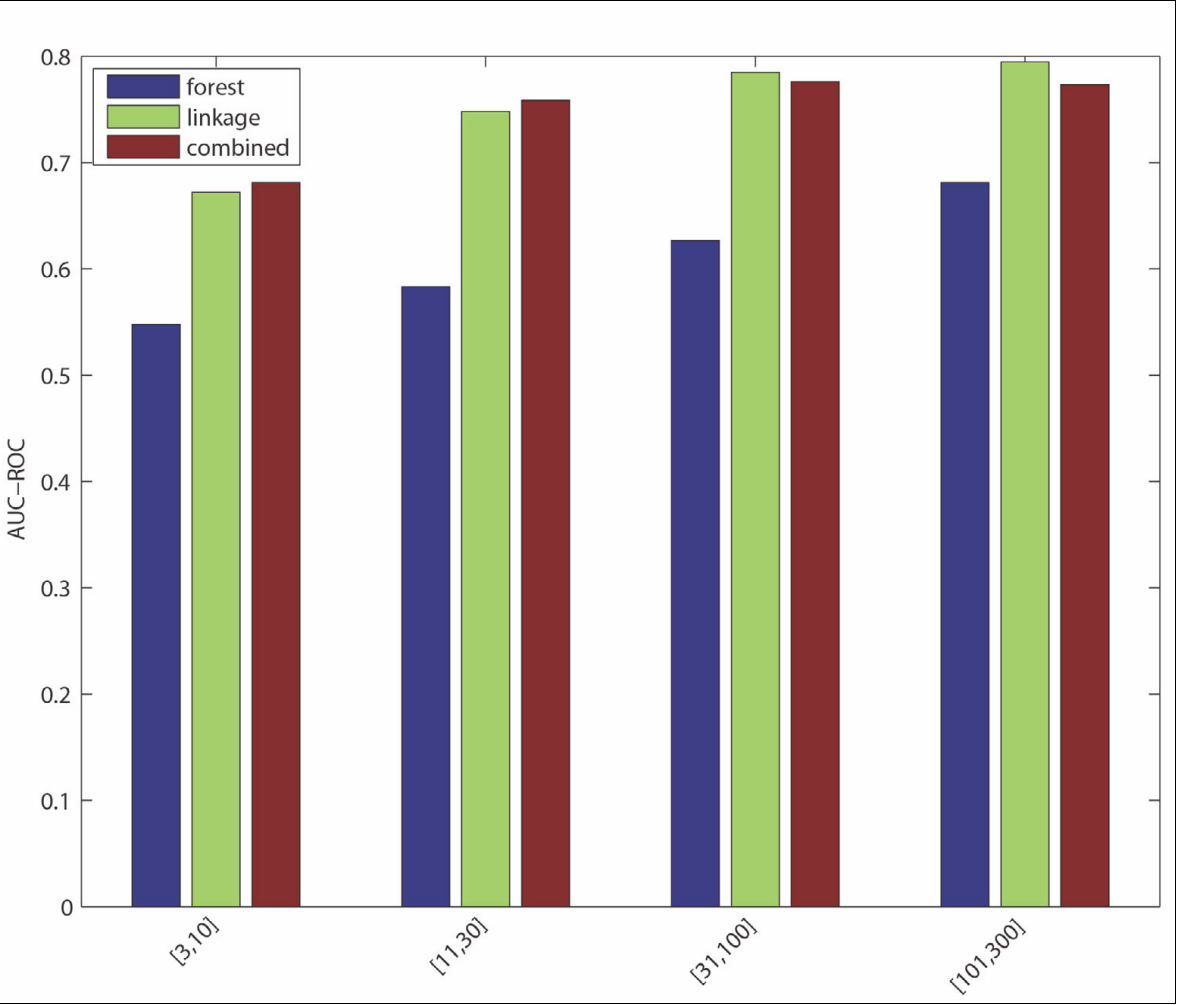
Mean MAP score across terms within each phenotype category. Note that in all categories the combined score provides a higher mean MAP. MAP, mean average precision.

As observed in [15], each 'base' classifier type (for example, functional linkage decision trees) has unique strengths in different categories and across different performance measures. The functional linkage approach ('guilt-by-association') tends to be the favored base classifier when evaluated by ROC-based measures (Figures 5 and 6), but the 'guilt-by-profiling' method excels when precision is the performance measure of choice (seen in Figure 7 and to a lesser extent in Figure 8). We believe that for many researchers the precision at low recall levels is of greatest importance - and is ultimately how the optimized combination method was guided in this study, aiding in the prioritization of a few high-scoring results for a laborious literature-based follow up.

As seen with *S. cerevisiae *[15], different base classifiers may each excel for some categories, or within a bounded specificity range of a single category (for example, seen in some cases in Figures 1 and 3). For prediction of *M. musculus *GO terms, Figure 2 shows that CC terms seem to benefit the most from using the functional linkage approach and that MF terms do not gain much from the integration of the functional linkage data. In contrast, MF annotation predictions (often relying on specific domain features - inherently gene-centric data - as primary predictors) tend to rely on the output of the random forests approach. By tailoring the data most appropriate for each style of base classifier, the combination approach leads to greater overall performance [15].

Figures 1, 3, 7, and 8 show the increase in precision across the entire recall range (and as a single MAP score) when using the combined scores relative to performance of each base classifier alone. Because we optimize the combination such that AUC-PR is maximized, we also checked to see whether the MAP measure for individual terms also increased. Figures 7 and 8 show that the mean of the MAP within each category is increased using the combined approach. This is consistent with results for *S. cerevisiae *function prediction [15] but had not been demonstrated for mammals, for phenotype prediction, or using the MAP measure. The combined classifier's performance in a precision-recall statistic does not guarantee improvement in other performance measures. However, the combination optimized for AUC-PR also led to AUC-ROC improvements across most GO categories (Figure 2) and had little impact for phenotype categories (Figure 6). We believe that predictions with the most utility are those with high precision, which tend to be at low recall. Thus, we conclude that the substantial benefits gained in precision using our regression-based precision-optimized combination easily justify any potential drop in AUC-ROC measures. The ability to increase predictive power using a combination of two classifiers leads to the natural question of, 'why stop at 2?' Indeed, one can combine in a similar fashion many different classifiers, with the number of degrees of freedom in the optimization problem increasing linearly with the number of base classifiers. Comparison of the optimized multiplicative coefficients might then be used to rate the relative contribution of each classifier to the final combined model, while simultaneously providing the best performance possible under the constraints of the method for combination chosen.

### A method for characterizing high-scoring predictions

As discussed in [15], random forests offer an advantage over some alternative machine-learning methods (for example, support vector machines, or monolithic classification trees) in that they provide a reliable variable importance measure. This provides valuable insight into why a prediction was made, offering the researcher a first step in understanding the biochemical principles underlying the annotation. As an example, a class (i) prediction associating the gene *Plscr1 *(phospholipid scramblase 1) and the GO term 'cholesterol homeostasis' used the Interpro-catalogued protein domain pattern 'Proteinase inhibitor, hirudin/antistatin' as the primary predictor (statins play a role in cholesterol regulation through inhibition of HMG-CoA reductase activity).

## Conclusion

We have computed a confidence score for every pairwise combination of nearly 7,000 function or phenotype annotation terms and over 20,000 *M. musculus *genes. Our classification method exhibits high precision (between 41% and 92% for GO categories at 1% recall) and has been shown to make accurate novel predictions [14]. A selection of our highest ranking novel predictions have also been examined in further detail, revealing a high (>80%) precision and immediately aiding in the annotation of mouse genes. The entire collection of scores and variable importance rankings are available to the public through a web interface. Our combined classifier approach has shown to be more effective than either base classifier alone, suggesting that integration of data sources and classifier approaches can improve predictions of phenotypes and confirming in mammals conclusions about this function prediction method made previously in *S. cerevisiae *[15]. These results support the idea that improved quantitative annotations may come by combining scores from large multi-group efforts like MouseFunc.

## Materials and methods

### Data sources

For both GO and mammalian phenotype term annotation prediction, our training data consisted primarily of the datasets assembled and integrated for the MouseFunc project. While much of the data originates from outside of the mouse community, the curation and data integration (for example, mapping between human interactions and their mouse equivalents) was primarily taken from the Mouse Genome Database [4] as part of the organization of the MouseFunc project. Cases where data used here differed from that used in the MouseFunc project are made clear below.

#### Expression data

Three expression datasets were used, two from microarray experiments [30,31], and a third [32] using serial analysis of gene expression (SAGE) [33]. After obtaining the data as described in the MouseFunc project [14], we performed additional clustering using k-means [34] and agglomerative hierarchical [35] methods. Hierarchical clustering used both complete- and average-linkage variants, with cuts at various depths in the resulting dendrograms to produce a predetermined number of clusters. The resulting number of clusters for each of the three methods was one of: 10, 20, 50, 100, 200, 500, and 1,000 for microarray data, and one of 10, 100, and 1,000 for SAGE data. Cluster membership was encoded by a binary attribute matrix (3 × [2 × 1,880 + 1,110] = 14,610 columns, 21,603 rows) such that each variable (column) represents membership for a given cluster, and each binary entry indicates membership for a given gene/cluster combination.

For the microarray-based expression data a log_2 _transformation was applied to the data provided for the MouseFunc project. Post-transformation, genes that did not rise above baseline sufficiently in any tissue (at least 40 observations >0 for Zhang data, at least 40 observations >3.0 for Su data) were removed. Genes were also removed if they did not show sufficient signal variation across tissues (difference between maximum and minimum signals must exceed 1.0 for Zhang data and 3.0 for Su data). The Euclidean (*L*_2_-norm) distance function was used for these data.

SAGE data provided for the MouseFunc project were first normalized within each library by that library's sum, thus reducing library-specific effects. Libraries within a single major tissue were then averaged (for example, two 'kidney' libraries were averaged). The clustering methods employed the Canberra distance, which is appropriate for count-based (Poisson-like) data. This metric, defined as the sum of the element-wise ratios of the difference of the two expression profiles to their sum, tends to accentuate the effect of a given difference among profiles close to zero. There is evidence [36] that a distance metric that acknowledges the Poisson nature of a counting-based method (for example, SAGE) may result in more biologically relevant clusters.

#### Protein domain patterns

Matches to protein domain patterns from the Pfam [37] and InterPro [38] databases were as provided for the MouseFunc project [14]. These data were placed in matrices with each column representing a particular domain (5,405 for Interpro and 3,133 for Pfam) and each row representing one of the 21,603 genes.

#### Protein-protein interaction

Binary protein interaction data from OPHID [39] was obtained as described in the MouseFunc project [14]. These data was then clustered into complexes using MCODE [40] (using the default parameter settings), resulting in 88 clusters (minimum cluster size = 3, maximum cluster size = 97). Each cluster is represented by a column in a binary matrix, in a similar fashion to the data sets described previously.

#### Phenotype

The mammalian phenotype annotations made available for the MouseFunc project consisted of only the 33 top-level terms. For the GO predictions, these annotations were used as-is (again, in a binary matrix form). For the mammalian phenotype predictions, the entire phenotype term ontology was examined in its most recent form. Annotations were made based on data available from the Mouse Genome Database [4]. After filtering for only those with annotation count in the range [3,300], 3,914 terms remained. Again, a binary matrix representation was used for the classifiers.

#### Phylogenetic profiles

Two data sources (BioMart [41] and Inparanoid [42]) were used in binary form as provided for the MouseFunc project [14]. Each variable with such data represents a species, leading to a binary vector for each mouse gene describing the presence of a known homolog in each species available (18 for BioMart and 24 for Inparanoid).

#### Disease associations

Disease associations from Online Mendelian Inheritance in Man (OMIM) [43], as provided for the MouseFunc project [14], consisted of 2,488 known Mendelian traits, each represented by a column in matrix form as above.

#### GO term annotations

GO annotations obtained November 2006 (the later of the two GO downloads employed within the MouseFunc project) were used for training GO prediction models, and (in some cases) for training phenotype prediction models. There were 2,938 GO terms within the three branches (BP, MF, CC) having annotation count in the range [3,300].

### Training data organization for functional linkage approach

Training data for the machine learning algorithms were organized generally as described in [15], with descriptions specific to *M. musculus *data manipulation given below.

The Pearson product-moment correlation coefficient of expression data was computed for each gene-pair (for each of the three data sets). These continuous coefficients were then binned into five groupings, *E*_0.5_, *E*_0.6_, *E*_0.7_, *E*_0.8_, and *E*_0.9_; each group defined as:

*E_x_*={(*a*, *b*)|*ρ*_1_(*a*, *b*) ≥ *x*}

where *a *and *b *are genes, and *ρ*_1 _is the correlation coefficient function. This resulted in 15 binary attributes describing each gene-pair. The protein-protein interaction data were already provided in gene-pair format, with each positive edge resulting in a positive gene-pair for a single binary matrix column representation.

Protein domain pattern data were converted to gene-pair format using the Jaccard similarity coefficient. Let *A *and *B *represent sets of annotated domains belonging to (respectively) genes *a *and *b*. The Jaccard similarity coefficient is then defined as:

$$\rho_{2}(a,b)=\frac{A\cap B}{A\cup B}$$

Those gene-pairs (*a*, *b*) having *ρ*_2_(*a*, *b*) ≥ 0.9 were assigned membership in a single set. Each domain dataset (Pfam and Interpro) was processed using this method, resulting in two binary variables.

The phenotype and OMIM disease data were given the same treatment as that for the protein domain data, resulting in two additional binary variables. The phylogenetic data sets were processed identically to the protein domain data, except that only three final Jaccard thresholds were used (0.7, 0.8, and 0.9), resulting in 6 additional binary variables (3 each for Biomart and Inparanoid data).

Thus, a total of 26 distinct binary variables describing each gene-pair were used by the functional linkage classifier. For phenotype prediction, eight additional variables were included representing co-annotation of terms within the MF and CC annotation sets (four groupings each based on annotation counts in ranges [3,10], [11,30], [31,100], or [101,300]), leading to 34 binary variables. We excluded terms within the BP branch to avoid potential circularity because GO BP terms are often tightly related to phenotypes. The data were then organized into a matrix with genes as rows and the 34 predictive variables as columns.

### Training data organization for random forests approach

For the random forest base classifiers, binary gene-centric data were represented in a binary matrix form (each row representing one gene, each column representing a property associated with that gene). When predicting GO terms, the training matrix was composed of the protein domain data, the phenotype data (top level only), the phylogenetic profile data, and the disease data (for a total of 5,405 + 3,133 + 33 + 18 + 24 + 2,488 = 11,101 columns). For phenotype term prediction, the training matrix consisted of the same data used for GO prediction as above except without the phenotype data (for a total of 11,068 columns).

### Probabilistic decision trees for functional linkage approach

To generate functional linkage graphs, a single probabilistic decision tree [44] was built for each category (12 for GO prediction, 4 for phenotype prediction). For each tree, the response variable was whether or not the gene-pair shared an annotation of a specific term within that category or any category in the same branch but with smaller annotation count. This response variable is thus used as a proxy for the general concept of two genes being 'functionally linked'. For GO term prediction, training examples (gene-pairs) were filtered such that only those genes with some existing GO annotation were included in the gene set, leading to $\left(\begin{array}{c} 10542\\ 2 \end{array}\right)$ unique gene-pairs. No such filter was performed for the four phenotype functional linkage trees (giving $\left(\begin{array}{c} 21603\\ 2 \end{array}\right)$ gene-pairs). Decision tree training and classification was then performed as described in [15].

### Random forests approach

Random forests are used as described in [15], with parameter choices and organism-specific details as listed below. For each GO term prediction a forest of 300 trees was built, with a column sample size chosen as *v*/20 where *v *is the number of remaining variables to be sampled at a given node. For the phenotype predictions, 200 trees/forest and the same *v*/20 were used as parameters. Each term (2,938 GO and 3,914 phenotype) corresponds to a separate random forest model.

### Logistic combination of methods

The two base classifiers for each GO or phenotype term were combined via a logistic model, as described in [15]. The *α *parameter corresponding to each category is shown in Table 3.

**Table 3:** Optimal logistic regression combination coefficients (*α *parameters) for each category

	BP	CC	MF	Phenotype
[3,10]	0.74	0.58	0.78	0.62
[11,30]	0.70	0.58	0.82	0.52
[31,100]	0.74	0.54	0.88	0.46
[101,300]	0.56	0.44	0.88	0.44

BP, biological process; CC, cellular component; MF, molecular function.

### Calibration of scores

The combined scores go through a final calibration so that they more closely approximate posterior probabilities. The calibration method developed for this study was also applied in the companion paper describing the MouseFunc project [14], where it was desirable to calibrate scores produced by vastly different learning methods (for example, support vector machines versus probabilistic decision trees). Briefly, for each term *t *we convert gene *i*'s score *s*_*i *_to a new score:

$$s_{i}^{*}=\frac{L\cdot s_{i}}{L\cdot s_{i}-s_{i}+1}.$$

*L *∈ [0,1] is a free parameter chosen such that the following relation holds:

$$\text{count}(t)=\sum_{i=1}^{n}s_{i}^{*}$$

where count(*t*) is the number of annotations for term *t *(that is, the average scaled score will equal the prior probability of positive annotation for term *t*).

## Abbreviations

AUC-PR, area under the precision-recall curve; AUC-ROC, area under the ROC curve; BP, biological process; CC, cellular component; GO, Gene Ontology; MAP, mean average precision; MF, molecular function; MGI, Mouse Genome Informatics; OMIM, Online Mendelian Inheritance in Man; ROC, receiver operator characteristic; SAGE, serial analysis of gene expression.

## Competing interests

The authors declare that they have no competing interests.

## Authors' contributions

FR conceived the study, and MT, WT and FR conceived of the methods. MT and WT performed all code construction and program optimization. FG aided in data pre-processing and clustering and constructed the web interface. DH performed literature evaluations of predictions with guidance from JB. MT and FR drafted the manuscript. All authors read and approved the manuscript.

## Additional data files

The following additional data are available with the online version of this paper. Additional data file 1 is an Excel spreadsheet listing predictions, ratings, and evidence supporting novel GO term predictions. Additional data file 2 is an Excel spreadsheet listing phenotype term predictions and supporting evidence.

## Supplementary Material

Additional data file 1 Predictions, ratings, and evidence supporting novel GO term predictions. Click here for file

Additional data file 2 Phenotype term predictions and supporting evidence. Click here for file
